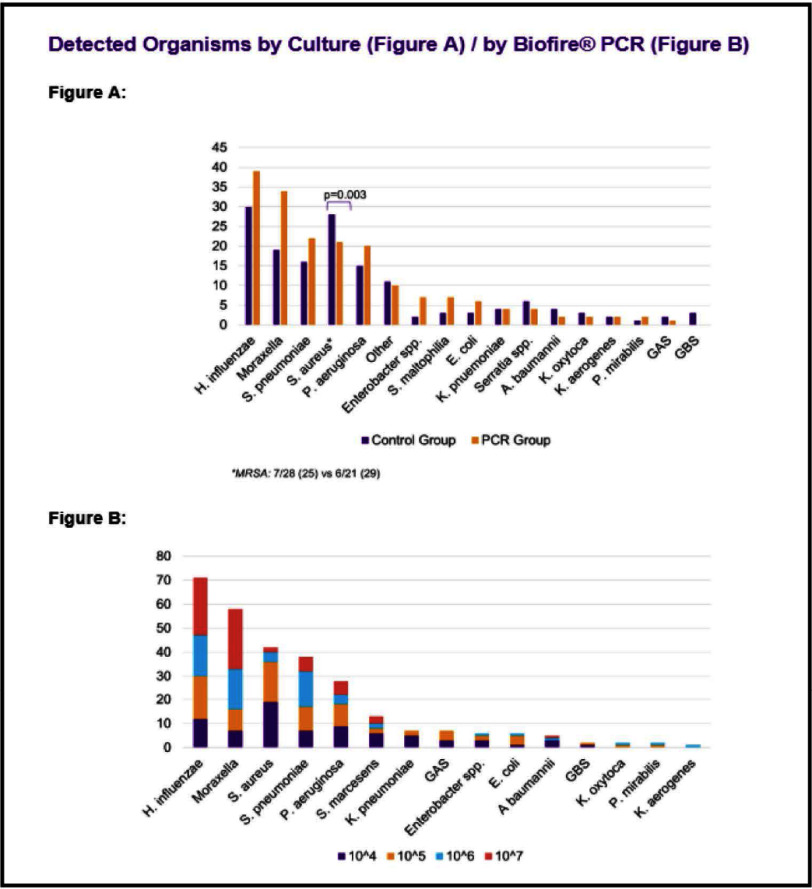# Impact of the Bronchoalveolar Lavage BioFire® FilmArray® Pneumonia Panel on Antimicrobial Utilization in Pediatric Patients

**DOI:** 10.1017/ash.2025.233

**Published:** 2025-09-24

**Authors:** Maria Celeste Ruiz Holgado, Kassandra Marsh, Anasemon Saad, Yanina Dubrovskaya

**Affiliations:** 1 NYU Langone Health; 2 NYU Langone Health, Hassenfeld Children’s Hospital

## Abstract

**Background:** Lower respiratory tract infections (LRTI) are a leading cause of pediatric mortality. Treatment typically involves empirical antibiotics, followed by de-escalation based on cultures, which are often negative. The BioFire® Pneumonia PCR panel detects pathogens rapidly, allowing for potentially faster optimization of antimicrobial therapy. Limited data exists on this panel in pediatrics, especially for bronchoalveolar lavage (BAL) samples. We hypothesized that patients with LRTI evaluated using the BAL PCR panel (PCR cohort) had shorter times to results and targeted therapy compared to those with BAL cultures alone (control cohort). **Method:** We conducted a retrospective study of patients aged 0–18 admitted to NYU Langone Manhattan with LRTI. The PCR cohort included patients from August 1, 2021, to November 1, 2023, who underwent both BAL culture and PCR testing. The control cohort included patients from August 1, 2017, to November 1, 2019, who had BAL cultures alone. The primary outcome was time to targeted therapy, defined as the time from culture collection to escalation or de-escalation. **Results:** A total of 280 patients were included (PCR n=172, Control n=108). The cohort had a median age of 3 years, with asthma (35% vs. 51%) and bronchiectasis (30% vs. 39%) being the most common comorbidities. Immunocompromised patients accounted for 7% of the PCR cohort and 6% of the control cohort. Most patients were on room air; 16% of the PCR group and 9% of the control group were intubated. Infectious Diseases was consulted more often in the PCR group (30% vs. 19%). PCR detected organisms faster compared to culture alone (4 vs. 25–26 hours). Discordant results between PCR and culture were 57%, with 81% due to additional bacteria detected by PCR. Stenotrophomonas maltophilia was the most common organism detected by culture but not included in the PCR panel. Time to targeted therapy was significantly shorter in the PCR group compared to culture group (0 vs. 1 day, p=0.003). Time to de-escalation was numerically faster in the PCR compared to control group (2 vs. 3 days, p=0.061). Fewer PCR patients received MRSA agents (34% vs. 55%, p=0.001). Rates of escalation, prior antibiotic use, and adverse outcomes were similar. **Conclusion:** The BioFire FilmArray Pneumonia Panel provides faster results and may aid in optimizing therapy in pediatric patients with LRTI.

**Figure:**
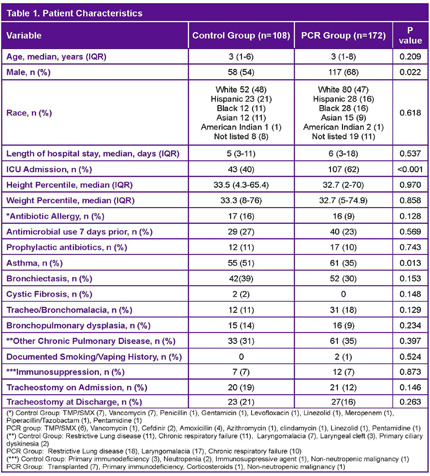

**Figure:**
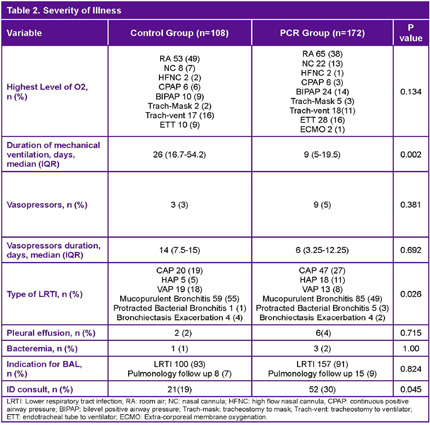

**Figure:**
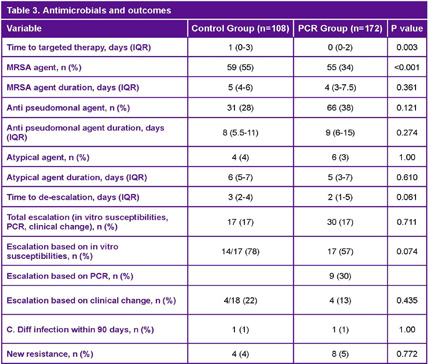

**Figure:**